# Performance Evaluation of CNT Reinforcement on Electroless Plating on Solid Free-Form-Fabricated PETG Specimens for Prosthetic Limb Application

**DOI:** 10.3390/polym14163366

**Published:** 2022-08-18

**Authors:** Palaiam Siddikali, P. S. Rama Sreekanth

**Affiliations:** School of Mechanical Engineering, VIT-AP University, Amaravati 522337, Andhra Pradesh, India

**Keywords:** PETG, MWCNT, electroless plating, tensile test, DMA, microhardness

## Abstract

The utility of polymers in the present decade is consistently increasing, giving scope to many applications from automobiles to prosthetics. Polymers used for solid free-form fabrication (SFFF), also known as 3D printing, comprise a quick fabrication process adopted by many industries to increase productivity and decrease the run time to cope with the market demands. In this research work, pure polyethylene terephthalate glycol (PETG) and multi-walled carbon nanotube (MWCNT)-PETG with an electroless metal layer coating and without a coating are discussed. The effect of the electroless metal layer coating on the reinforced PETG-MWCNT results in improved mechanical, tribological, and other surface properties. Pure PETG was incorporated with MWCNT nanofillers at 0.3 wt.% and extruded as a filament through a twin screw extruder with a 1.75 mm diameter and printed on ASTM standards. Tensile testing was performed on all four types of un-coated pure PETG, PETG-MWCNT, and metal-layer-coated PETG and PETG-MWCNT with a coating thickness of 26, 32, 54, and 88 μm. Dynamic mechanical analysis (DMA) showed that the coated PETG-MWCNT had the highest storage and loss modulus. The heat deflection temperature was improved to 88 °C for the coated PETG-MWCNT. The wear volume against the sliding distance at a load of 40, 50, and 60 N showed that the coefficient of friction decreased with an increase in the load. The scratch test results revealed the lowest penetration depth and lowest friction coefficient for the coated PETG-MWCNT sample. The water contact angle test showed that a greater coating thickness makes the sample surface more hydrophobic, and the microhardness test indicated that the indentation hardness value for the PETG-MWCNT was 92 HV. The study revealed that the metal-layer-coated PETG-MWCNT had better performance compared to the other specimens due to a good metal layer bonding on the PETG substrate. It was concluded that adding MWCNTs to a metal layer electroless coating improved the surface and mechanical properties of the PETG, and this may be suitable for many applications.

## 1. Introduction

Since the 1980s, 3D printing has grown in relevance for research, business, and enthusiasts. This technology allows for the production of complicated forms and products that would otherwise be impossible to make using traditional procedures in a relatively short time. Metallic products, on the other hand, may be created using direct metal processing techniques such as SLM and DMLS or by traditional casting foundry processes [1]. Both of these approaches are difficult to implement if the primary goal is to keep material costs low and production simple. In the case of a prosthesis for limb loss patients, the design of the parts is user-specific and cannot be standardized [2]. Therefore, the alternative method for achieving a metal finish at a faster and lower cost is by 3D-printed components and then metalizing only the polymer’s surface, which provides the desired metal characteristics on polymers/composites while reducing the weight. Physical vapor deposition (PVD) may not always be preferred due to its cost, although metallization is based on wet chemistry. Many of the polymers used in SFFF, such as ABS, PLA, PET, PETG, and others, can be readily metalized utilizing an electroless plating technique [3], allowing for uniform and thick layers of coating on 3D-printed samples [4]. Copper (Cu), nickel (Ni), and a few other metals are potential coating materials. Polylactic-acid- (PLA) [5] and polyethylene-terephthalate-glycol-modified (PETG) polymers are easier to print because of their better dimensional stability during solidification [6]. Therefore, the PETG material was chosen for further processing with multi-walled carbon nanotubes.

Nanofillers are categorized based on their size, geometry, composition, and aspect ratio [7]. CNTs, typically 0.1 to 5% by weight [8], can not only improve the mechanical and thermal characteristics, but also provide electrical conductivity to the polymer [9]. In the present work, PETG was reinforced with multi-walled carbon nanotubes (MWCNTs), as they show better mechanical properties and, hence, are of considerable interest. The tensile strength of multi-walled carbon nanotubes is around 50–200 MPa, whereas Young’s modulus is above 800 MPa [10]. Another main aspect of processing the polymers is the nanofillers’ dispersion; this can be performed through various methods such as melt intercalation, in situ polymerization, and solution intercalation [11]. Furthermore, the incorporation of in situ photo polymerizations with 3D printing technology can benefit the preparation of polymer nanocomposites with a variety of macroscopic sizes and shapes [12].

Electroless coatings can be utilized for both aesthetic and functional purposes. Specific emphasis is given to the plating of stiff, durable substrates created by the SFFF method, with applications in the field of prosthetics and orthotics. Electroless plating enables the metalizing of 3D objects with simpler equipment by lowering the cost and boosting overall efficiency [13]. This technique excludes the need for costly vacuum processing and complicated equipment; it also enables flexible substrate metallization. 

Researchers have worked on PETG samples and their composites fabricated through the fused deposition technique. Szykiedans et al. [14] investigated the mechanical performance of PETG and PETG reinforced with glass fibers produced using the fused deposition modeling technique or 3D printing, and the researchers discovered that PETG with glass fiber reinforcement performed better than non-reinforced PETG. Hamidi et al. [15] used FDM technology to present printability findings for PETG reinforced with carbon nanotubes (CNTs). They discovered a significant degree of distortion in PETG prints without CNTs as compared to PETG+CNT, which demonstrated strong bonding within the print and resulted in a low void content. Sofiane et al. [16] used universal testing equipment to investigate the tensile performance of PETG filaments. Tensile tests were performed at a set displacement rate of 5 mm/min until the filament ruptured. It was reported that Young’s modulus and the tensile strength were 2.01 GPa and 66 MPa, respectively. To the best of the authors’ knowledge, no study has been reported on the electroless metal layer coating of PETG-MWCNT. The present study shows how the electroless metal-coated PETG-MWCNT’s mechanical and tribological parameters differ from uncoated PETG-MWCNT. 

## 2. Materials and Methods

### 2.1. Materials

**Figure 1:**
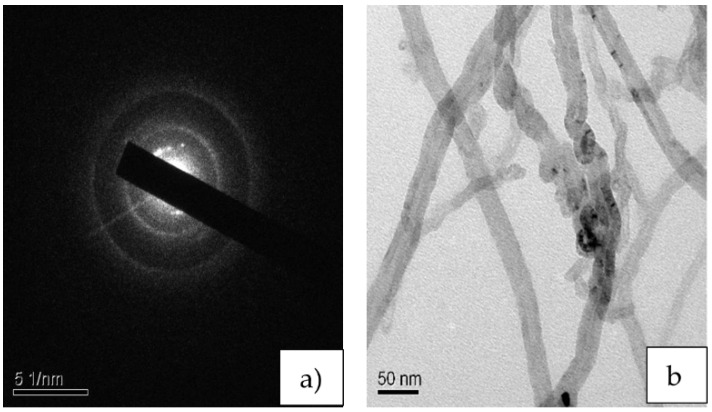
(**a**) SAED pattern; (**b**) TEM of CNTs.

The polyethylene-terephthalate-glycol-modified (PETG) pellets were a polyester-based material provided by Nexgen Plasstek (Iyava-Sanand, Ahmedabad, Gujarat, India). The PETG pellets had a diameter of roughly about 2 ± 0.05 mm. MWCNTs were functionalized as detailed by Dalai et al. [17]. Selected area diffraction (SAD) of the functionalized MWCNTs is shown in Figure 1a. The ring structure indicates the semi-crystalline nature of the MWCNTs after chemical functionalization [18]. The randomly oriented MWCNTs are shown in Figure 1b [19,20,21]. The multi-walled carbon nanotubes were purchased from M/s NanoShel Pvt. Ltd. (Sundran, Punjab, India). The reinforcement of PETG with 0.3 wt.% MWCNTs was achieved by coating the MWCNTs through the solvent evaporation method, followed by blending and filament formation through a twin screw extruder. The obtained filament was 1.75 mm in diameter and was used as the filament for 3D printing.

### 2.2. Methods

The fabrication of the composite sample was performed as follows. MWCNTs at 0.3 wt.% were dispersed in ethanol using ultrasonication for about 30 min. The PETG pellets were then added to this solution and heated on a hotplate and simultaneously stirred. MWCNTs were coated on the PETG samples by the solvent binding technique along with the ultrasonication process, as discussed by Satya et al. [22]. In the next step, the pellets were subjected to a twin screw extrusion (Aasabi Twin screw extruder, India) process to obtain the desired diameter of the filament wire, and after extruding the filament wire, it was used for the 3D printing technique as discussed below.

#### 2.2.1. Fabrication of 3D-Printed Specimens

**Figure 2:**
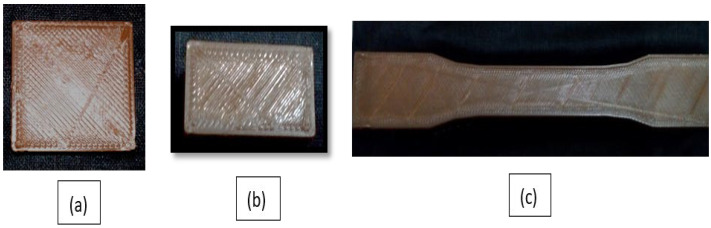

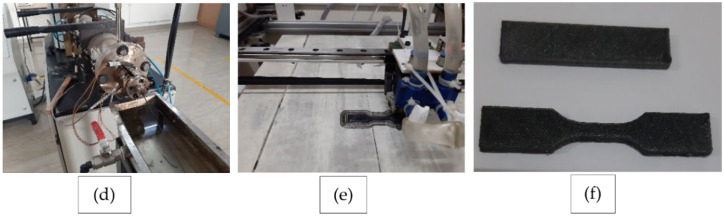
(**a**–**c**) show pure PETG 3D-printed samples based on the ASTM standards; (**d**) twin screw extrusion of the 3D printing of the PETG filament; (**e**) 3D printing of the PETG+MWCNT tensile specimen; (**f**) final printed PETG+MWCNT specimen.

The 3D printing process was undertaken using a commercial FDM machine. Composites were characterized for their strength using a fused filament fabrication machine based on the ASTM D638 standard [23]. All the pure PETG and PETG-MWCNT samples were fabricated using the “Pramaan Platinum SFFF machine” (Additive manufacturing India Pvt. Ltd., Bengaluru, India). For proper adherence, the construction platform was wrapped with painter’s tape (2093EL tape Scotch Blue, 3M). The individual thickness of the layer was set to 0.3 mm with an infill density of 100% for the 3D structure. A 220 °C to 265 °C extrusion temperature and a 60 °C bed temperature were used to print the PETG-MWCNT filament substrate. Figure 2a–c below show the pure PETG sample, and Figure 2d shows the filament being extruded through the twin screw extruder machine; Figure 2e,f show the tensile specimen during printing and the final printed tensile sample.

#### 2.2.2. Electroless Copper Metallization of 3D-Printed PETG-MWCNT Samples

First, during the activation stage, conditioner was applied to the surface of the samples of the PETG-MWNCT, followed by an etching process for 5–15 min, in a bath with a temperature range of 60–65 °C, to encourage more uniform absorption. Etching was followed by neutralizing with sodium bisulfite by careful elimination of surplus acid and other contaminants. After this, the samples were subjected to copper activation, which was performed by introducing a liquid precious metal activator with a low concentration for about 3 min at 45 °C. The PETG and PETG-MWCNT samples were submerged in a copper electroless solution following the photo-reduction process [24]. The catalytic sites, which served for the adsorption of the copper ions, were Ag-NPs. The deposition of copper metal on the catalytic substrate was due to the transfer of an electron to the silver seeds of the adsorbed copper ions [25]. The entire reaction for electroless copper deposition using formaldehyde (HCHO) as a reducing agent is shown in Equation (1).

Cu^2+^ + 2HCHO + 4OH^−^ → Cu + 2HCOO^−^ + 2H_2_O + H_2_ (1)

**Figure 3:**
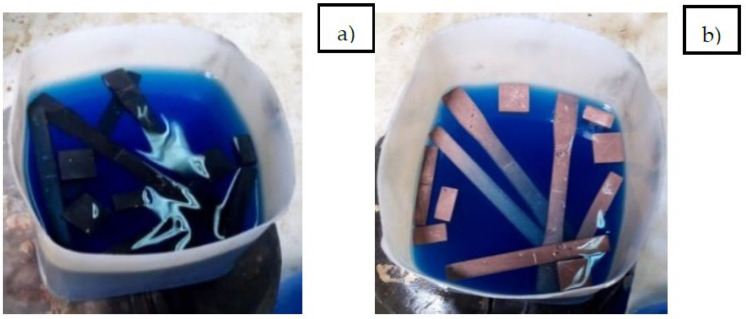
(**a**) PETG+MWCNT specimen activation stage; (**b**) copper deposition on the PETG+MWCNT specimens.

**Figure 4:**
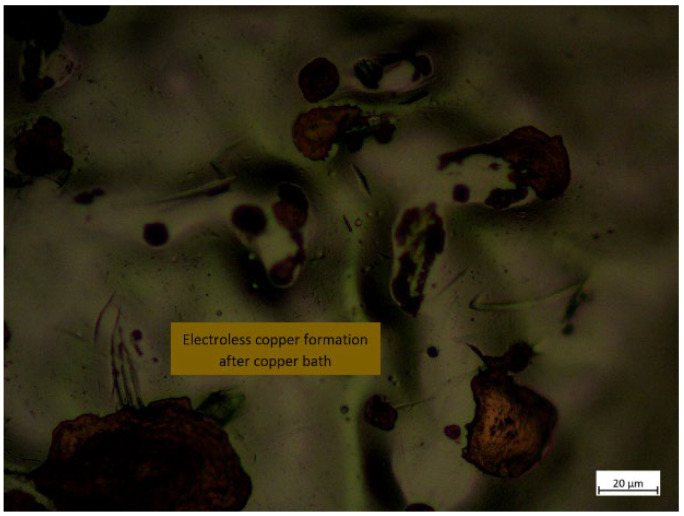
Copper formation during the electroless process on the PETG+MWCNT specimen in microscopic view.

The deposited copper acted as seed layers for further adsorption of copper ions, and the procedure was repeated. Then, for accuracy, the specimens were tested under a microscope to determine whether the copper was properly deposited on the surface or not. The entire copper deposition process is shown in Figure 3a,b, and the microscopic view of the specimen after copper activation is shown in Figure 4.

**Figure 5:**
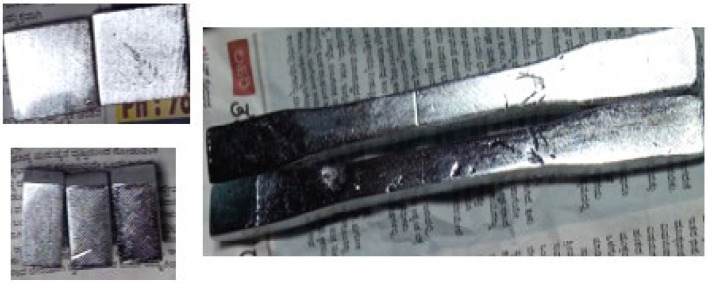
Electroless nickel-plated PETG+MWCNT specimens.

After copper deposition on the PETG+MWCNT specimens, the specimens were dipped in a nickel bath to make the plastic surface conductive. In most applications, nickel is the metal of choice. However, because copper is less resistant to scorching, it is occasionally used in automobile parts, but proper reinforcement of MWCNTs in PETG followed by a nickel bath can be a useful choice for prosthetic-limb-related applications. The electroless plating of copper and nickel deposition thickness on the specimen surface after 4 h, 8 h, 16, and 24 h were approximately 26 μm, 32 μm, 54 μm, and 88 μm, respectively. The final nickel-plated PETG+MWCNT specimens are shown in Figure 5.

#### 2.2.3. Tensile Test

To identify the characteristics of the fabricated samples, a tensile test was performed using a Tinius Olsen 10 KL universal testing machine in the temperature range of 22 to 24 °C according to the ASTM D638 standard. A maximum strain rate of 50% was allowed for the specimens. Based on the original sample dimension, the load–displacement curves were constantly recorded, and the stress–strain curves were plotted and the obtained results reported.

#### 2.2.4. Dynamic Mechanical Analysis 

To understand the response of the fabricated and coated samples under dynamic loading at different temperatures, dynamic mechanical analysis (DMA) of the samples was performed. A Perkin Elmer DMA 8000 (Winter Street, Waltham, MA, USA) was used in the current study. A temperature scan was performed from 30 °C to 120 °C at a rate of increment of 5 °C/min in three-point bending mode. The sample dimensions were 49 mm × 13 mm × 3 mm, and the tests were performed according to the ASTM D4065 standard.

#### 2.2.5. Heat Distortion Temperature 

The heat distortion test (HDT) indicates the temperature at which the polymer materials deform under mechanical stress when subjected to a specific fiber loading. The HDT was conducted on samples based on the ASTM D648 standard using an HDT tester (SC Dey & Co., Kolkata, West Bengal, India). The test samples were immersed in silicone oil, and the temperature was gradually increased by 2 °C. During the procedure, a force of 0.45 MPa was given to the samples at a 0.25 mm deviation of the specimen. The test was intended to understand the influence of the electroless metal layer coating and MWCNT reinforcement on the heat distortion temperature of the PETG.

#### 2.2.6. Wear Test

The specimens for the sliding wear test were prepared on SFFF with a 100% infill density. The wear test was performed based on the ASTM G 99 standard on a Ducom, TR-201 POD Tribometer pin-on-disk under dry sliding conditions at room temperature. The specimens obtained from extrusion followed by fused deposition and electroless plating interacted against a stainless steel 316 L counterface. Wear analysis on the samples was monitored at different loading conditions of 40 N, 50 N, and 60 N for a sliding distance of 1.2 km at a sliding velocity of 1.2 m/s at 600 RPM. The wear rate, wear volume, and coefficient of friction were used to analyze the wear performance of the fabricated samples.

#### 2.2.7. Water Contact Angle Measurements (Wettability Test)

The surface’s wettability was measured according to its contact angle (θ). The coated and uncoated PETG+MWCNT specimens were analyzed by a liquid droplet of deionized water. A 5 µL drop was placed onto the test sample by a microsyringe dispenser. The wettability test through contact angle measurement helps to determine the balance of adhesive and cohesive forces acting on the contact line.

#### 2.2.8. Scratch Test

**Figure 6:**
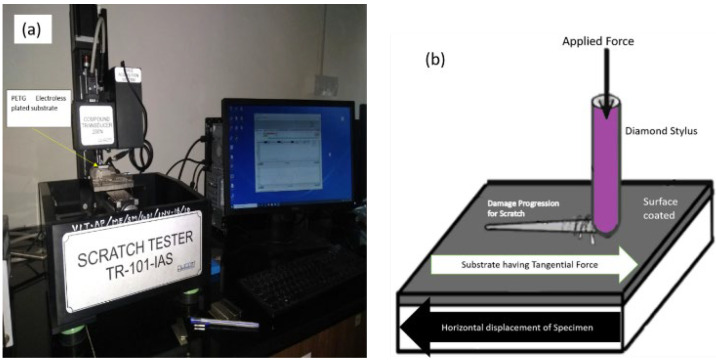
(**a**) Scratch adhesion test machine, (**b**) schematic view.

The adhesive strength between the coated layer and PETG/PETG-MWCNT substrate determines the effectiveness of the electroplating. The scratch depth and the mechanism involved and the scratch friction coefficient are important parameters to assess the coating’s adhesive strength. The parameters used for performing the scratch test are as given in Table 1 below, and the scratch testing machine is shown in Figure 6.

The indentation depth and force of friction were monitored with the indenter movement for all scratch tests to estimate the cracks’ critical loads, delamination, chipping, failure of the coating, and other phenomena. When experiments at multiple loading rates gave almost equal results and changes in the loading rate had little effect, the rate of loading for scratch testing was set at 30 N/min. As the scratch progressed, observations such as top layer peeling, a pile-up on the sidewalls, the visibility of small fractures to long wide fissures inside the coatings, chipping, porosity, and partial or entire coating delamination were revealed.

#### 2.2.9. Microhardness

A three-sided pyramid micro-Vickers hardness indenter was used to determine the hardness of the electroless plated and pure PETG substrates. This test was performed at a steady load of 300 g with a 5 s holding time. Five readings were taken on each sample, and the average value of hardness is reported.

## 3. Results and Discussion

### 3.1. Tensile Strength

**Figure 7:**
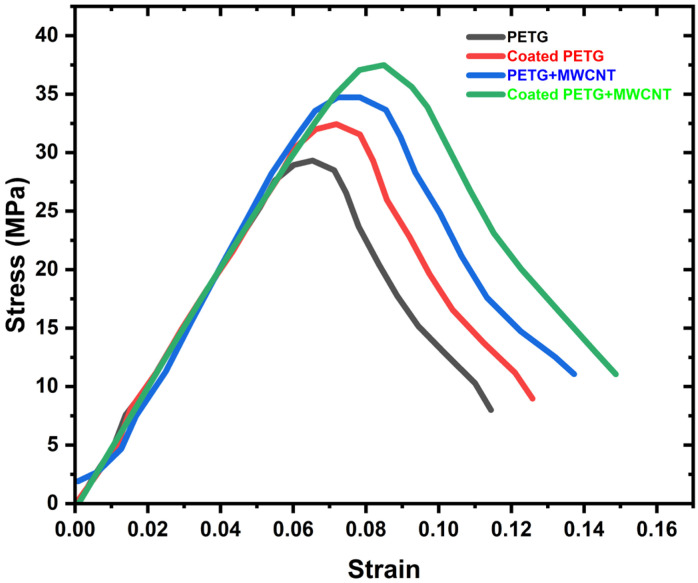
Stress–strain curve for various specimens.

The stress–strain behavior of the PETG, coated PETG, PETG+MWCNT, and coated PETG+MWCNT samples are shown in Figure 7. The figure reveals that the curves have a linear nature up to the yield point. Later, the curves have a nonlinear nature, and the maximum stress was observed for the coated PETG+MWCNT, i.e., 37.72 MPa. The stress sequence of different PETG samples is as follows: coated PETG+MWCNT > PETG+MWCNT > coated PETG > PETG, with maximum stress values of 34.83 MPa for PETG+MWCNT, 32.12 MPa for coated PETG, and the smallest value of 28.96 MPa for pure PETG, as mentioned in the graph.

**Figure 8:**
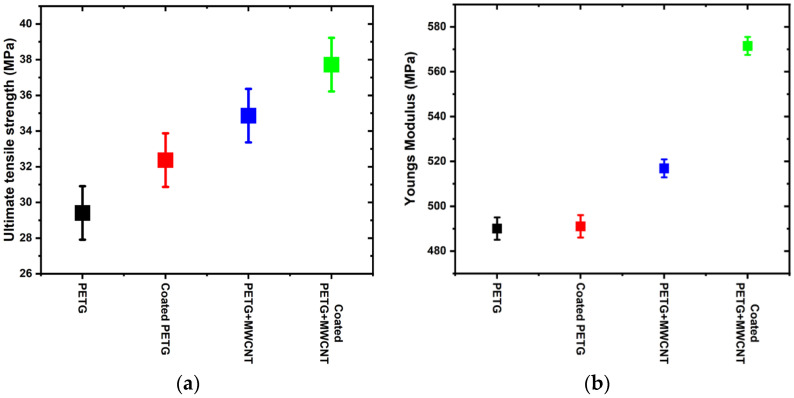
(**a**) Ultimate tensile strength of various tensile samples; (**b**) Young’s modulus of PETG specimens.

The ultimate tensile strength of various PETG samples is shown in Figure 8a. The addition of MWCNTs improved the strength of the PETG. An increment of 9%, 15.6%, and 22% was observed in the coated PETG, PETG+MWCNT, and coated PETG+MWCNT, respectively, when compared to pristine PETG. The ultimate tensile strength (UTS) sequence of different PETG samples was as follows: coated PETG+MWCNT > PETG+MWCNT > coated PETG > PETG. The improved mechanical qualities were due to better bonding between the matrix and filler, as well as the ability of long-chain creation with no agglomeration. Pore reduction associated with CNT concentration is another element that contributed to the tight connection between phases.

The Young’s modulus of the PETG specimens is shown in Figure 8b. The figure depicts that the addition of MWCNTs improved the modulus of the coated PETG+MWCNT sample as compared to the pure PETG. The Young’s modulus of a material indicates its stiffness and is obtained as the ratio between the stress and strain at the proportionality limit. The amount of strain produced in a material is directly proportional to the stress acting on it up to the proportionality limit, beyond which there is a permanent deformation. The Young’s modulus of various samples is reported as obtained from the software Tinius Olsen, 10 kN UTM. The Young’s modulus of pure PETG was observed to be 490 MPa, and electroless plating did not considerably increase Young’s modulus. The reason for this is quite obvious: Young’s modulus is a bulk material property, and electroless plating influences only the surface property, not the bulk material property. It was also observed that adding MWCNTs to PETG increased the modulus to 516 MPa, and the coating and MWCNTs together improved Young’s modulus further to 570 MPa. The effect of coating on Young’s modulus was negligible for the pure PETG samples, whereas for the MWCNT-reinforced samples, the coating increased the modulus. The reason for this can be attributed to the metallic coating, which firmly adhered to the PETG-MWCNT sample.

The UTS and Young’s modulus are shown in Table 2, which shows the ultimate tensile strength and Young’s modulus for PETG, coated PETG, PETG+MWCNT, and coated PETG+MWCNT; for pure PETG, the UTS was 29.41 MPa, whereas the coated PETG showed little improvement, 32.37 MPa. Similarly, MWCNT-reinforced PETG showed a greater value of 34.86 MPa, while the highest ultimate tensile strength of 37.72 MPa was observed for the coated PETG+MWCNT; this indicates that the coating on the MWCNT-reinforced PETG can make the polymer material stronger with a reduced weight, which can be used for various applications.

### 3.2. Dynamic Mechanical Analysis 

**Figure 9:**
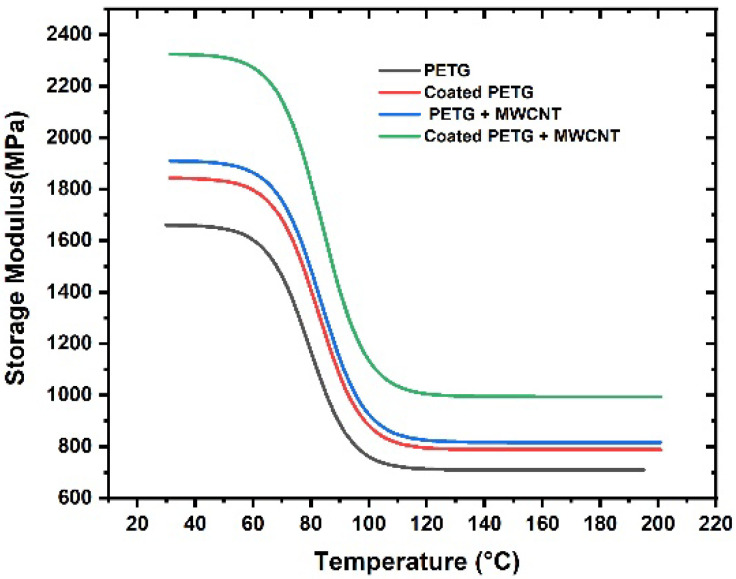
Storage modulus vs. temperature.

**Figure 10:**
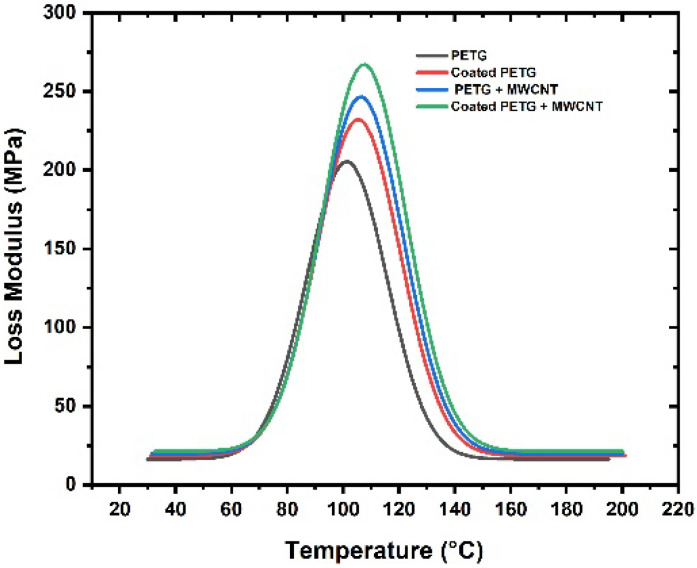
Loss modulus vs. temperature.

**Figure 11:**
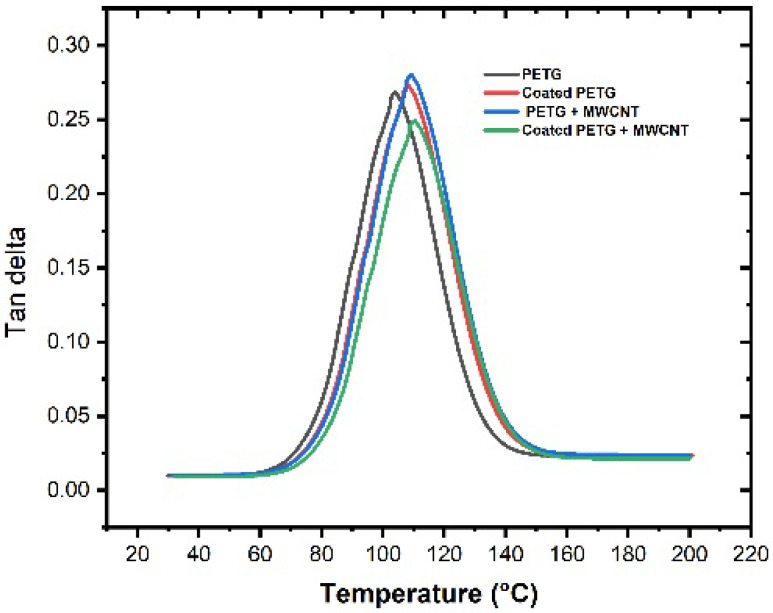
Tan delta vs. temperature.

The storage modulus of all samples was observed within the temperature range of 30 °C to 200 °C, as shown in Figure 9. At a temperature of 37 °C, the storage modulus of the pure PETG substrate was 1625 MPa, which increased by 11%, 15%, and 30% for the coated PETG, PETG-MWCNT, and coated PETG-MWCNT. The highest storage modulus was observed for the coated PETG-MWCNT sample: 2300 MPa at 37 °C; it increased by 30% in comparison with the PETG sample and 19% compared with the coated PETG sample. The possible reason for the increment in the storage modulus is the good internal adhesion of Cu+Ni with the MWCNT fillers in the PETG matrix due to the uniform absorption of the metal ions in the matrix [26]. Furthermore, the strong energy storing capacity of MWCNTs during deformation due to the applied load resulted in a high storage modulus for the coated PETG-MWCNT sample [27]. Similarly, at 36 °C, the loss modulus was observed as 201 MPa for the pure PETG and further increased by 13%, 20%, and 35% for the coated PETG, PETG+MWCNT, and coated PETG+MWCNT. The highest loss modulus was observed for the coated PETG-MWCNT substrate at 270 MPa, as shown in Figure 10. This is due to the highly conductive nature of Cu and Ni, leading to the excitement of molecules, which results in the dissipation of energy due to the friction developed between the internal molecules with an increase in the temperature, as well as the rubbery nature and flow behavior of the PETG matrix with the increase in temperature, leading to a decrement in the storage and loss moduli [28]. The damping nature of the samples was studied at a temperature sweep of 30 °C to 200 °C, and it was observed that, at 30 °C, all the samples had an elastic behavior with minimal internal molecular movement, resulting in no dissipation of energy. From the graph shown in Figure 11, as the temperature increased, the tanδ increased and suddenly decreased in the temperature region of 110–120 °C, indicating more energy dissipation with increasing temperature due to high polymer chain mobility in the matrix and the high conductive nature of Cu and Ni coatings, followed by high frictional moments in the MWCNT particles with the surrounding matrix and absorbed metal ions.

### 3.3. Heat Distortion Test

**Figure 12:**
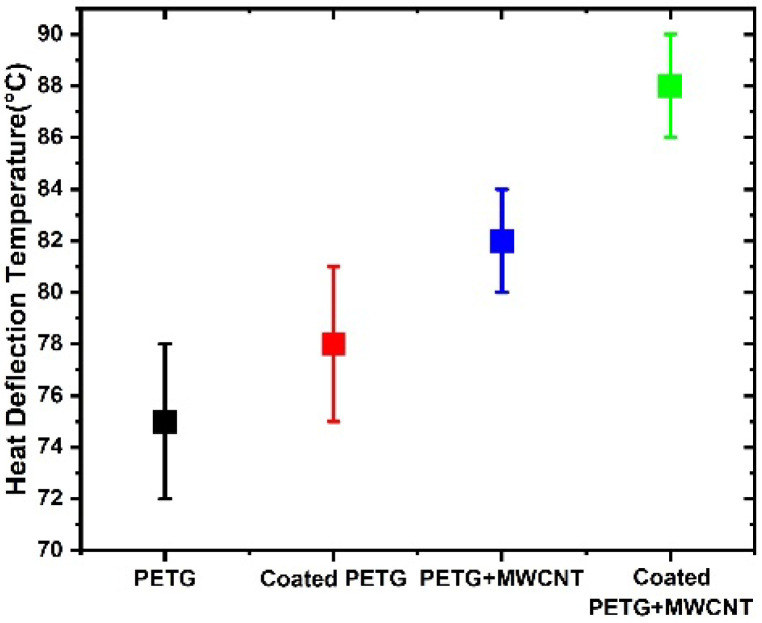
Heat deflection temperature for various PETG specimens.

The heat distortion temperature represents the polymer’s resistance to deflection at a certain temperature and load. This is a crucial characteristic for determining a polymer’s maximum operating temperature in practical applications. According to the trend, increasing the MWNCT filler concentration results in a rise in the HDT value, as shown in Figure 12. There was a 13% increase in the HDT value at 0.3% (by weight) loading. In general, the introduction of nanofillers impedes molecular chain mobility, causing the HDT value to increase [29]. This is related to decreases in crystallinity, changes in the glass transition temperature of that composite, as well as the reinforcing impact. Heat distortion values in the figure show that the pure PETG without coating had a temperature of 75 °C, while the coated PETG showed an improvement of around 78 °C, due to the metallic coating being a good conductor of heat; when the heat distortion temperature rises, the coated PETG will be the first heat recipient, and after that, it is conducted to the inside PETG. After adding MWCNTs to the PETG, the values show that the HDT value increased to 82 °C. Further, in the case of the coated PETG+MWCNT with metallization, there was a greater increase in the HDT value to 89 °C. The coated specimen showed a 15% improvement in the HDT value, and it can sustain a greater heat distortion temperature when compared to the uncoated PETG-MWCNT specimens, making the specimen more suitable for various heat-related applications.

### 3.4. Wear

**Figure 13:**
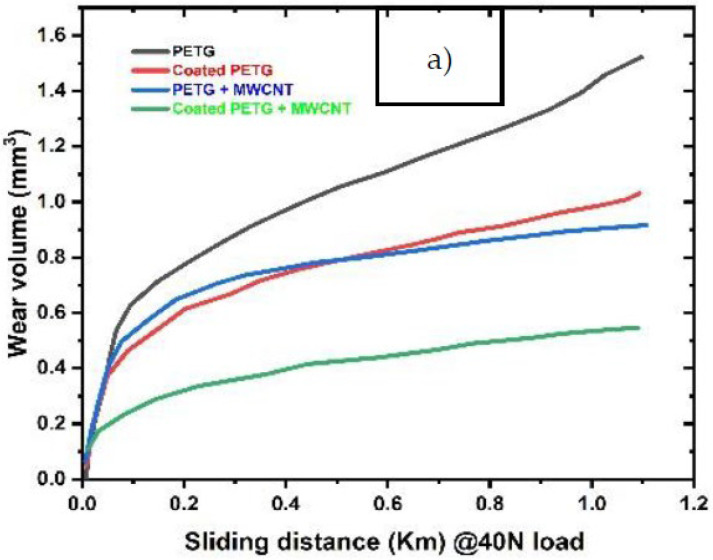

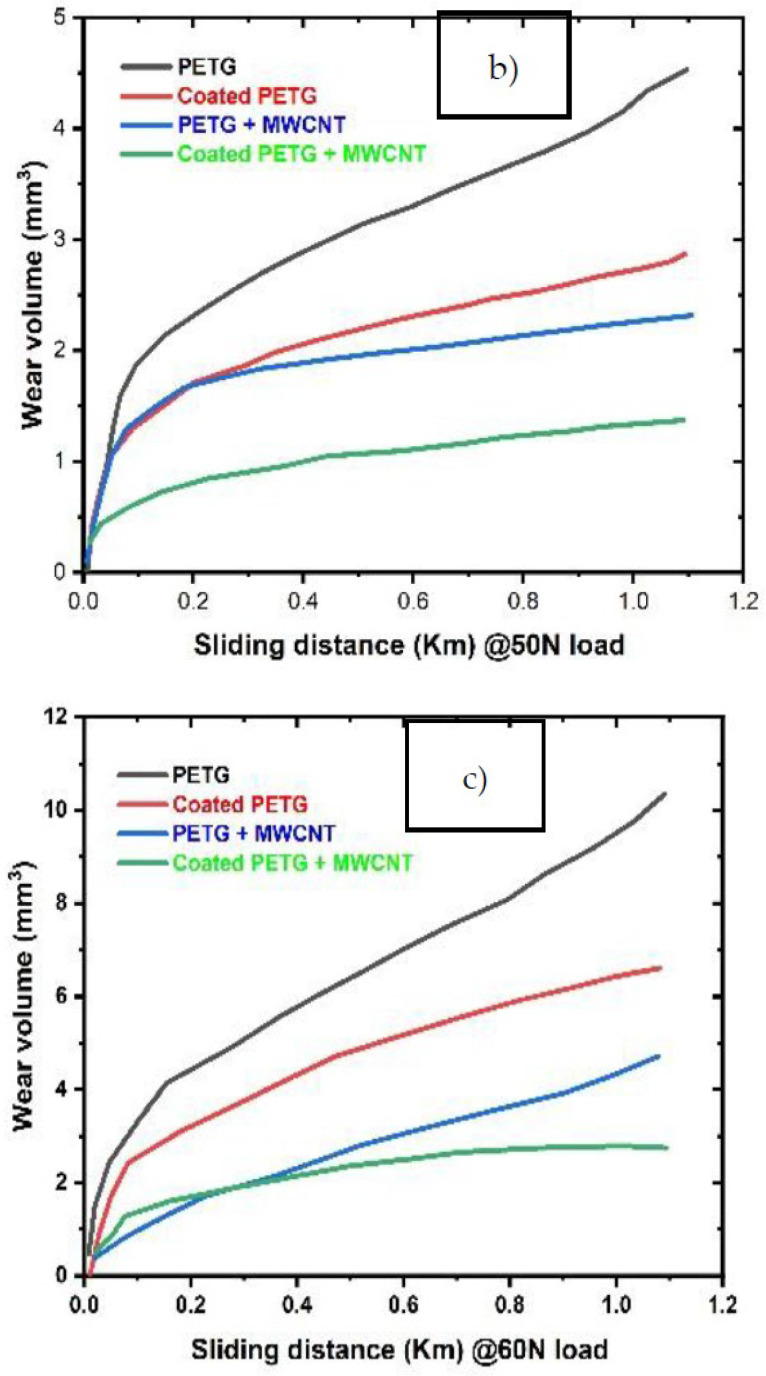
Wear volume for the sliding distance at: (**a**) 40 N load; (**b**) 50 N load; (**c**) 60 N load.

**Figure 14:**
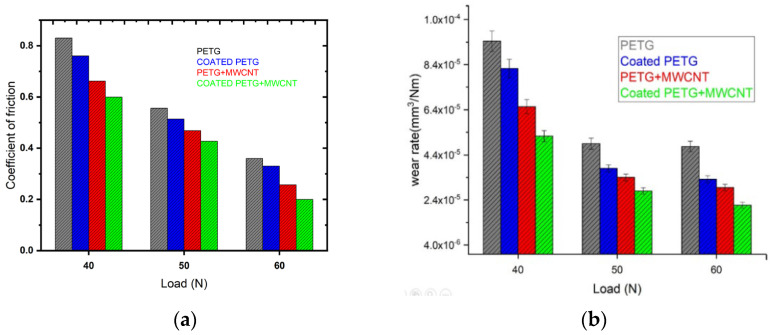
(**a**) Wear rate at different loads; (**b**) coefficient of friction at different loads.

A pin-on-disc test was employed to study the wear behavior of the samples. Figure 13 shows the wear volume of all four samples, PETG, PETG+MWCNT, coated PETG, and coated PETG+MWCNT, against sliding distance. The total wear volume obtained is related to the linear wear at steady state conditions. Initially, the wear volume of the samples was high and nonlinear due to the non-flat surfaces. After achieving a flattened surface, the wear volume tended to increase with the sliding distance linearly. It was observed that the wear volume of the coated PETG-MWCNT was the lowest at all loading conditions due to an increase of the hardness with the presence of Cu and Ni particles associated with the MWCNTs, leading to a reduction of wear. From Figure 13a–c, it is observed that the PETG sample had the highest wear volume of 1.52 mm^3^, 4.51 mm^3^, and 10.9 mm^3^ at 40 N, 50 N, and 60 N loads and the lowest for the coated PETG-MWCNT with 0.51 mm^3^, 1.34 mm^3^, and 2.21 mm^3^ at the same loads. The wear rate of all the samples represents the material worn per one joule of work performed. From Figure 14a, it is observed that the wear rate of pure PETG was 9.35 × 10^−5^, 4.98 × 10^−5^, and 4.89 × 10^−5^ mm^3^/Nm at 40 N, 50 N, and 60 N loads and reduced to 4..28 × 10^−5^, 2.56 × 10^−5^, and 2.38 × 10^−5^ when reinforced with MWCNTs and coated; this is due to the strong interfacial adhesion between the MWCNTs and surrounding particles, leading to a strong bond between the layers, resulting in an increment in the hardness and a high work to failure ratio. It was observed that the coefficient of friction decreased with an increase in the load for all the samples, as shown in Figure 14b; the coefficient of friction of the pure PETG was high at 0.82, 0.56, and 0.38, whereas, for the coated PETG-MWCNT, it was 0.6, 0.45, and 0.2.

### 3.5. Contact Angle

**Figure 15:**
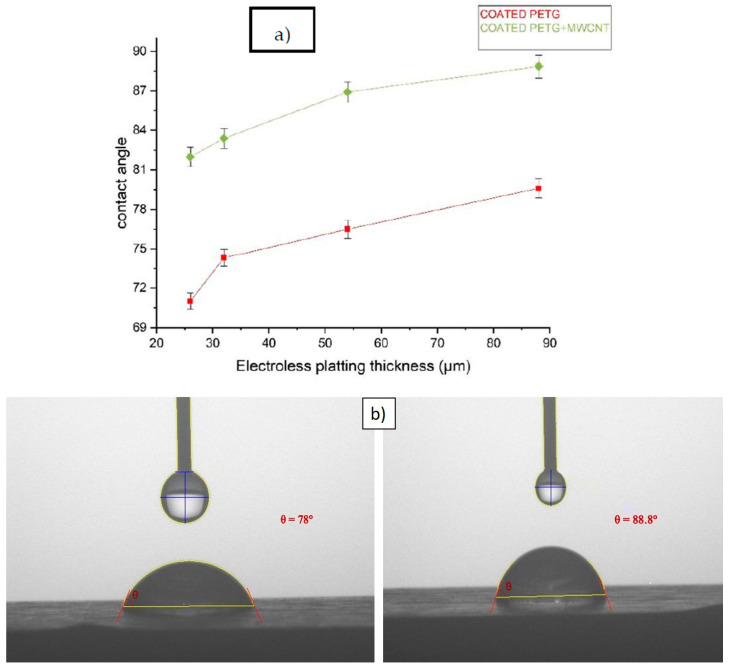
(**a**) The contact angle for various electroless plating thicknesses; (**b**) water droplet contacting the specimen’s surface.

Figure 15 illustrates the water contact angle for the coated PETG and coated PETG-MWCNT specimens with varying thicknesses of the electroless plating. The equipment was fixed with a charge-coupled device (CCD) camera to monitor and capture photos of the contact angle of the liquid with the surface interface of the specimen. When the adhesive forces exceed the cohesive forces, the liquid drop wets the surface (θ less than 90°), but when the cohesive forces exceed the adhesive forces, the surface is just partly wet. Water is commonly used as the reference liquid; hence, when the contact angle of a water droplet on a surface is acute, the surface is said to be hydrophilic, whereas when contact angles of θ are greater than or equal to 90°, the surface is said to be hydrophobic. The contact angle (θ) of the liquid drop put on the solid substrate is used to determine the wettability of a solid surface associated with a certain liquid. The greater the contact angle, the less wettable the surface. The liquid’s cohesive forces reduce the surface area per volume, causing the droplet to ball up and avoid further contact with the surface. Adhesive forces, on the other hand, tend to optimize the solid–liquid contact area depending on the chemistry of the substrate and its interaction with the liquid [30].

The water contact angle for the electroless plated PETG specimen was 71.2, 74.6, 76.2, and 78 degrees for the thicknesses of 26 μm, 32 μm, 54 μm, and 88 μm, and for the same electroless plated PETG-MWCNT thickness specimen, the contact angle was at 82, 83.4, 86.91, and 88.84 degrees, respectively, as shown in Figure 15a. The contact angle of the coated PETG-MWCNT specimen was high when compared with the coated PETG because of the presence of the MWCNTs associated with the PETG, which showed a positive effect, as shown in Figure 15b. It was observed that both samples had a hydrophilic nature, causing the surface to have more wettability with the presence of dominant adhesive forces. By this water contact angle test, it was evident that without MWCNTs, the water absorption on the surface of the specimen was more flattened, such that we can assume the specimen may absorb, but for the PETG-MWCNT, it showed that the water was more curved on the surface of the specimen, showing that the inclusion of MWCNTs will make the surface non-wettable, because when the contact angle increases, wettability decreases, which in turn does not affect the wear behavior of the specimens.

### 3.6. Scratch Test

**Figure 16:**
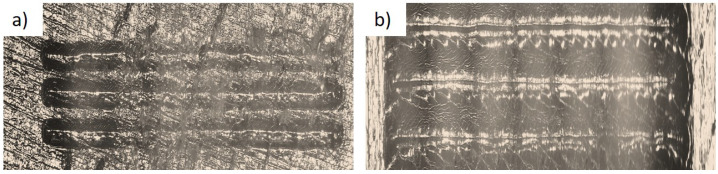
Scratch test for the electroless plated PETG+MWCNT specimen (**a**) under the scratch test machine, microscopic view, and (**b**) expanded view under the scratch tester.

Analyzing scratch properties is essential in exploring the suitability of coatings for tribological applications. The scratch hardness, penetration of depth, and scratch coefficient of friction were studied with the load varying from 2–30 N with a scratch velocity of 1 mm/s over the coated and non-coated surfaces. The width of the scratch was measured by a microscope, as shown in Figure 16a,b, to evaluate the scratch hardness, using Equation (2).

**Figure 17:**
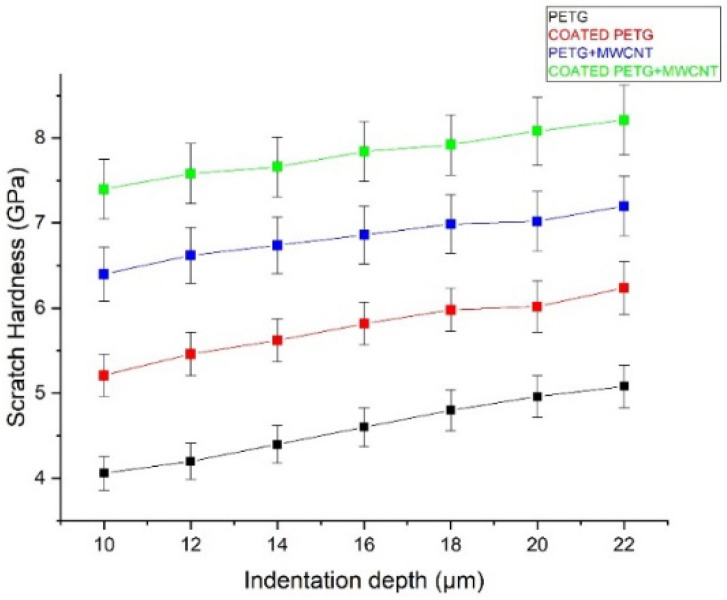
Scratch hardness vs. indentation depth.

The scratch hardness values of the PETG, PETG-MWCNT, coated PETG, and coated PETG-MWCNT samples were obtained at different values of the penetration depth of the applied loads. It can be observed that the Cu-Ni-coated PETG-MWCNT contributed to an improvement in the hardness, as it opposed the displacement of the indenter and prevented deformation when compared with other samples [31]. From the results, it is noted that the scratch hardness of the PETG, coated PETG, PETG+MWCNT, and coated PETG+MWCNT specimens was 4.6 MPa, 6 MPa, 6.8 MPa, and 8 MPa, as shown in Figure 17 [32].

(2) $$\mathrm{Scratch}\;\mathrm{hardness}=\frac{8\times P}{\pi \times w^{2}}$$

where:

*P* = applied load;
*w* = width of the scratch measured from the microscope;
$\frac{8}{\pi }$ = constant value.

**Figure 18:**
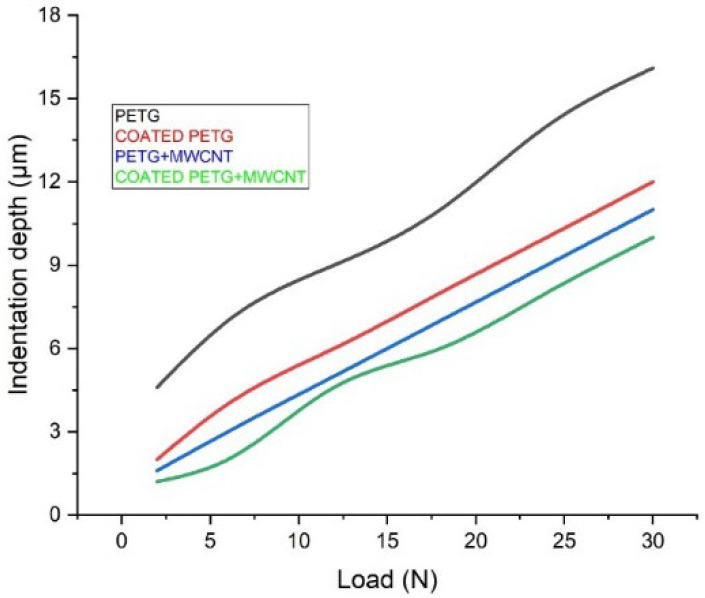
Indentation depth vs. load.

At a load of 30 N, the coated PETG-MWCNT samples had a penetration depth of 10 μm for various scratch velocity rates. The pure PETG sample had a penetration depth of 16 μm, and for the PETG-MWCNT and coated PETG, the penetration depth was observed to be 11 μm and 12 μm, as explained in Figure 18. The variation in specimen properties influenced the depth of penetration. These results may be compared to those obtained for the PETG-MWCNT substrates coated with Ni to determine whether the coating is comparatively superior in terms of abrasion resistance. However, factors such as the substrate’s natural slope (variation of the thickness of the substrate) and mounting issues might increase the scope of error in these tests. During the scratch testing of the coatings, the cohesive critical loads on (CL1) and adhesive failures of (CL2) the PETG+MWCNT specimens were 2–14 N and 16–30 N, respectively.

**Figure 19:**
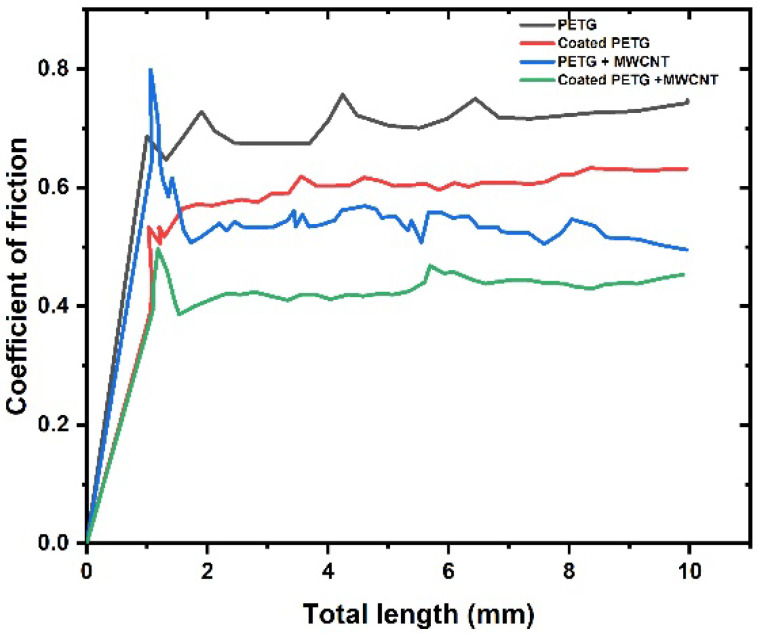
Coefficient of friction vs. total length.

The friction coefficient (μ) was found to rise as the load increased along the length, which reflects the rate for the coating and substrate combination, as mentioned in Figure 19. From the test, it was observed that the pure PETG sample had a high coefficient of friction of 0.8, followed by the coated PETG sample with 0.69, due to a lack of adhesive forces between the layers. The coefficient of friction for the PETG-MWCNT specimen was 0.52, followed by the coated PETG-MWCNT specimen with 0.49; due to the strong hardness of the material, causing high resistance to the indenter, this caused deformation [33].

### 3.7. Microhardness

**Figure 20:**
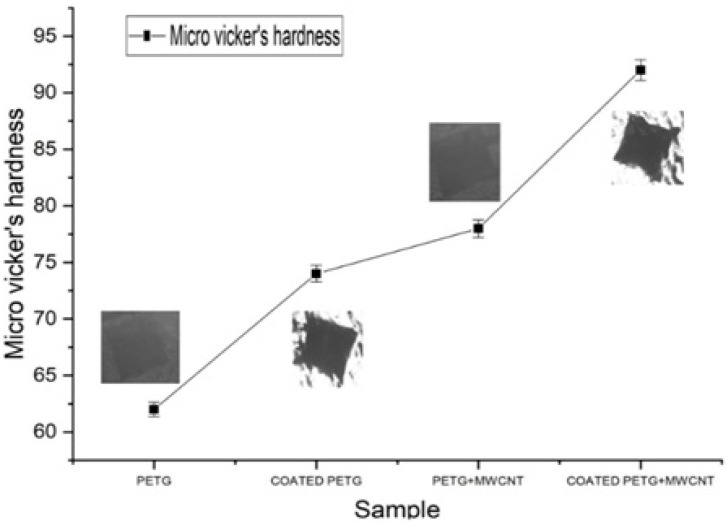
Micro-Vickers hardness for various PETG samples.

A three-sided pyramidal micro-Vickers hardness indenter was used to determine the hardness of the electroless plated and non-electroless plated substrates. The indentation hardness of the coated composite substrate was the highest at 92 HV. Parameters such as the amount of coating deposition, reinforcements, and their distribution pattern change the hardness values of the samples [34]. Particle strengthening and dispersion strengthening are the two factors that vary the microhardness capacity of the material. The uniform dispersion of reinforcements and coated embedments into the matrix provides the dispersion strength by avoiding the formation of agglomerations [35]. Varying the thickness of the embedments and the amount of reinforcement provides strength to the particles and helps transfer the load between embedments, reinforcements, and matrix. In this study, we observed that the electroless plated samples had noticeably high hardness values by improving the particle strength and offering resistance to plastic deformation. In this work, the pure PETG had a hardness value of 63, which further increased to 18% at 74 for the coated PETG; similarly, the PETG+MWCNT had a hardness value of 76, a 23% increment, and the coated PETG-MWCNT had a 53% increment, with the highest harness value of 92, as shown in Figure 20.

## 4. Conclusions

In this work, PETG was blended with MWCNTs, and electroless metal layer coating on 3D-printed PETG and PETG-MWCNT specimens was successfully carried out. The mechanical characteristics were investigated using the tensile test, dynamic mechanical analysis, heat distortion test, and wear and scratch test measurements, including contact angle and microhardness test parameters. The addition of MWCNTs to the PETG specimens with electroless metal layer coating showed superior properties when compared to the uncoated pure PETG. The conclusions from the present work are as follows:

- With the addition of MWCNTs to the PETG, there was an improvement in the base material properties. Furthermore, the electroless metal layer coating enhanced the strength of the PETG material.
- The heat distortion temperature value increased due to the coating on the PETG-MWCNT compared to the uncoated PETG sample, showing that the material can resist high temperatures; hence, the blended PETG+MWCNT polymer with the coating can be extensively used for various applications.
- The wear characterization specified that the initial wear rate was considerably decreased due to the thickness of the electroless metal layer coating on the PETG-MWCNT specimen, and through the contact angle measurement, it was evident that, with a greater coating thickness on the PETG-MWCNT specimen, the wettability was maximum.
- From the scratch test, it was noted that the PETG-MWCNT substrates coated with Ni had the lowest penetration depth and lowest friction coefficient. The microhardness test indicated the high indentation hardness and, hence, proved that it is superior in terms of abrasion resistance.

Hence, it is concluded that an electroless metal layer coating on PETG reinforced with MWCNTs would significantly enhance the thermal and mechanical properties, which are of primary importance for various applications including, prostheses.

## Figures and Tables

**Table 1:** Scratch number, load, stroke length, scratch velocity, and scratch offset tabulated values for the scratch test.

Scratch No.	Load (N)	Stroke Length (mm)	Scratch Velocity (mm/s)	Scratch Offset (mm)
1	10.0	10	1.0	0.50
2	20.0	10	1.0	0.50
3	30.0	10	1.0	0.50

**Table 2:** Ultimate tensile strength (UTS) and Young’s modulus of various test samples.

Material	UTS	Young’s Modulus (MPa)
PETG	29.41	490.03
Coated PETG	32.37	491.04
PETG+MWCNT	34.86	516.87
Coated PETG+MWCNT	37.72	571.5

## Data Availability

Not applicable.
